# A transgenic mouse line for collecting ribosome-bound mRNA using the tetracycline transactivator system

**DOI:** 10.3389/fnmol.2014.00082

**Published:** 2014-10-29

**Authors:** Laurel Drane, Joshua A. Ainsley, Mark R. Mayford, Leon G. Reijmers

**Affiliations:** ^1^Department of Neuroscience, School of Medicine, Tufts UniversityBoston, MA, USA; ^2^Graduate Program in Neuroscience, Sackler School of Graduate Biomedical Sciences, Tufts UniversityBoston, MA, USA; ^3^Department of Molecular and Cellular Neuroscience, The Scripps Research InstituteLa Jolla, CA, USA

**Keywords:** TRAP, tetracycline transactivator, Camk2a, Fos, CA1 pyramidal neuron

## Abstract

Acquiring the gene expression profiles of specific neuronal cell-types is important for understanding their molecular identities. Genome-wide gene expression profiles of genetically defined cell-types can be acquired by collecting and sequencing mRNA that is bound to epitope-tagged ribosomes (TRAP; translating ribosome affinity purification). Here, we introduce a transgenic mouse model that combines the TRAP technique with the tetracycline transactivator (tTA) system by expressing EGFP-tagged ribosomal protein L10a (EGFP-L10a) under control of the tetracycline response element (tetO-TRAP). This allows both spatial control of EGFP-L10a expression through cell-type specific tTA expression, as well as temporal regulation by inhibiting transgene expression through the administration of doxycycline. We show that crossing tetO-TRAP mice with transgenic mice expressing tTA under the Camk2a promoter (Camk2a-tTA) results in offspring with cell-type specific expression of EGFP-L10a in CA1 pyramidal neurons and medium spiny neurons in the striatum. Co-immunoprecipitation confirmed that EGFP-L10a integrates into a functional ribosomal complex. In addition, collection of ribosome-bound mRNA from the hippocampus yielded the expected enrichment of genes expressed in CA1 pyramidal neurons, as well as a depletion of genes expressed in other hippocampal cell-types. Finally, we show that crossing tetO-TRAP mice with transgenic Fos-tTA mice enables the expression of EGFP-L10a in CA1 pyramidal neurons that are activated during a fear conditioning trial. The tetO-TRAP mouse can be combined with other tTA mouse lines to enable gene expression profiling of a variety of different cell-types.

## INTRODUCTION

Acquiring the gene expression profiles of individual cell-types is imperative for understanding their molecular identities. Although traditionally cell types are defined by their morphology and functional properties, recent studies have shown that many subclasses of neurons can also be distinguished through genome-wide gene expression profiling (Okaty et al., 2011). These studies used various techniques to collect mRNA from defined groups of neurons, including laser capture microdissection (LCM) and fluorescence-activated cell sorting (FACS). However, both LCM and FACS-based methods have various drawbacks. LCM often results in low levels of mRNA, and is prone to contamination from the close proximity of off-target cell-types (Okaty et al., 2011). In addition, LCM requires fixation of the tissue which alone can increase degradation of nucleic acids. Use of manual cell dissociation techniques can eliminate the off-target contamination seen with LCM. However, this technique can be very time consuming as large numbers of cells must be isolated in order to collect sufficient mRNA quantities. FACS greatly increases the number of cells that can be isolated. However, the harsh conditions of the dissociation and sorting process causes neurons to lose their processes and increases the risk of introducing gene expression artifacts (Okaty et al., 2011).

Many of the technical limitations of traditional mRNA collection methods can be circumvented with the translating ribosome affinity purification (TRAP) technique (Doyle et al., 2008; Heiman et al., 2008, 2014), and other methods based on collecting epitope-tagged ribosomes (Sanz et al., 2009). The TRAP technique utilizes transgenically expressed EGFP-L10a (enhanced green fluorescent protein fused with the ribosomal protein L10a) to affinity purify EGFP-tagged ribosomes from genetically defined cell-types. Through analysis of the co-purified mRNA, it is possible to acquire the translational profile of the cell-type in which EGFP-L10a was expressed. TRAP has been applied to cell-types in various mouse tissues including brain (Doyle et al., 2008; Heiman et al., 2008; Schmidt et al., 2012; Ainsley et al., 2014), heart (Fang et al., 2013; Zhou et al., 2013), liver (Wilkins et al., 2014), and kidney (Liu et al., 2014). In addition to mice, TRAP has been applied in other species such as drosophila and zebrafish (Thomas et al., 2012; Huang et al., 2013; Tryon et al., 2013). TRAP has the advantages of being more high-throughput than LCM without requiring the dissociation of neurons from intact tissue as needed for FACS. In addition, the ribosome-bound mRNA collected through TRAP provides a more accurate representation of cellular protein content than the total mRNA collected through LCM or FACS (Schwanhausser et al., 2011).

Here, we introduce a novel, versatile transgenic mouse model for the spatial and temporal control of EGFP-L10a expression, thereby increasing the number of cell-types amendable to TRAP analysis. We achieved this by placing expression of EGFP-L10a under control of the tetO promoter. The tetO promoter is activated by tetracycline transactivator (tTA), which can be temporally suppressed through addition of doxycycline (dox) to the food of the mice (Gossen and Bujard, 1992). As a result, temporally controlled cell-type specific gene expression can be achieved by breeding tetO mouse lines with mouse lines that express tTA under cell-type specific promoters. Accordingly, the tTA system has been used in a wide variety of studies that required spatial and temporal control of transgene expression (Schonig et al., 2010), including studies on the neural correlates of memory storage (Mayford et al., 1996; Reijmers et al., 2007). Here we crossed the tetO-TRAP mouse with both Camk2a-tTA and Fos-tTA driver mice, thereby generating Camk2a-TRAP and Fos-TRAP mice, respectively. In a recent study we used the Camk2a-TRAP mouse to collect ribosome-bound mRNA from dendrites of CA1 pyramidal neurons (Ainsley et al., 2014). Here we present a more extensive characterization of the EGFP-L10a expression patterns in the Camk2a-TRAP mouse, and demonstrate how it can be used to collect ribosome-bound mRNA from complete hippocampal CA1 neurons. In addition, we demonstrate with the Fos-TRAP mouse how the temporal control conferred by the tTA system enables the expression of EGFP-L10a in neurons activated during a defined time-window.

## MATERIALS AND METHODS

### TRANSGENIC ANIMAL CREATION AND BREEDING

All animal procedures were performed in accordance with the National Institutes of Health Guide for the Care and Use of Laboratory Animals and were approved by the Tufts University Institutional Animal Care and Use Committee. We generated a transgenic mouse expressing the ribosomal protein L10a tagged with EGFP under the control of the tetracycline operon (tetO-TRAP). The EGFP-L10a fusion protein coding sequence in a pLD53.SC.EGFP-L10a plasmid was donated by Nathaniel Heintz at Rockefeller University. The plasmid was transformed into One Shot PIR1 Chemically Competent *E. coli* (Life Technologies #C1010-10) and the EGFP-L10a sequence was obtained and verified using the services of the Tufts University Core Facility. A DNA fragment containing the EGFP-L10a coding sequence was removed by incubating the plasmid with AfeI and BamHI restriction endonucleases. After agarose gel electrophoresis, a ∼1.4 kb band containing the EGFP-L10a coding sequence was cut from the gel and purified using the PureLink Quick Gel Extraction Kit (Life Technologies #K2100-12). This fragment was treated with T4 DNA Polymerase (NEB #M0203S) to form blunt ends. The blunt end EGFP-L10a fragment was cloned into pMM400Sfi, a plasmid for transgenic mouse creation containing a tet operon (donated by Mark Mayford at the Scripps Research Institute). pMM400Sfi was digested with the EcoRV restriction endonuclease to create blunt ends and then treated with Calf Intestinal Alkaline Phosphatase (Life Technologies #18009-019) to dephosphorylate the blunt ends and prevent self-ligation. The blunt end EGFP-L10a fragment was cloned into linearized pMM400Sfi by incubating with T4 DNA Ligase (NEB, #M0202S) overnight at 16°C. The correct sequence and insert orientation was verified by DNA sequencing.

Pronuclear microinjection of pMM400Sfi.EGFP-L10a into C57BL/6J embryos was performed at the Tufts Medical Center Transgenic Core Facility. Potential transgenic founder lines were screened for transgene integration using the following genotyping primers: (5′-CAGAACGCACACCGAGAACT-3′, 5′-CTTCAAGGACGACGGCAACT-3′). Founders with detectable transgene integration were crossed with C57BL/6J mice to ensure heritability. Offspring carrying the transgene were crossed to Camk2a-tTA transgenic mice (JAX Stock Number 003010) to induce transgene expression in Camk2a-positive neurons in the forebrain. Individual founder lines were screened for strong, specific expression matching the expected spatial pattern and one line was used for all further experiments. This tetO-TRAP transgenic mouse has been submitted to the Jackson Laboratory for distribution (JAX Stock Number 024898).

### FEAR CONDITIONING

To ensure that mice remained in a resting state with minimal neuronal activation prior to fear conditioning (FC), all animals were singly housed and left undisturbed for at least 3 days prior to the start of FC. This enabled us to control neuronal activation with high temporal specificity.

#### Camk2a-TRAP mice (**Figure 3**)

Male and female, 2–3 month old mice were singly housed for at least 3 days prior to behavioral testing. On day 0, mice were submitted to one contextual FC trial. FC trials were performed in a specialized chamber (Coulbourn Instruments; H10-11RTC, 120W × 100D × 120H) and consisted of 500 s with 2 s long, 0.7 mA foot shocks administered at 198, 278, 358, and 438 s.

#### Fos-TRAP mice (**Figure 5**)

Male and female, 3–4 month mice were singly housed for at least 5 days prior to behavioral testing. On day-3, dox was removed from the food. On day 0, mice were then either left in their cage until tissue dissection (home cage, HC), or submitted to three contextual FC trails each spaced three hours apart. At the end of the third FC trial, all Fos-TRAP mice (HC and FC) were returned to a diet containing dox.

### TISSUE PREPARATION FOR IMMUNOHISTOCHEMISTRY AND IMAGE COLLECTION

**FIGURE 1:**
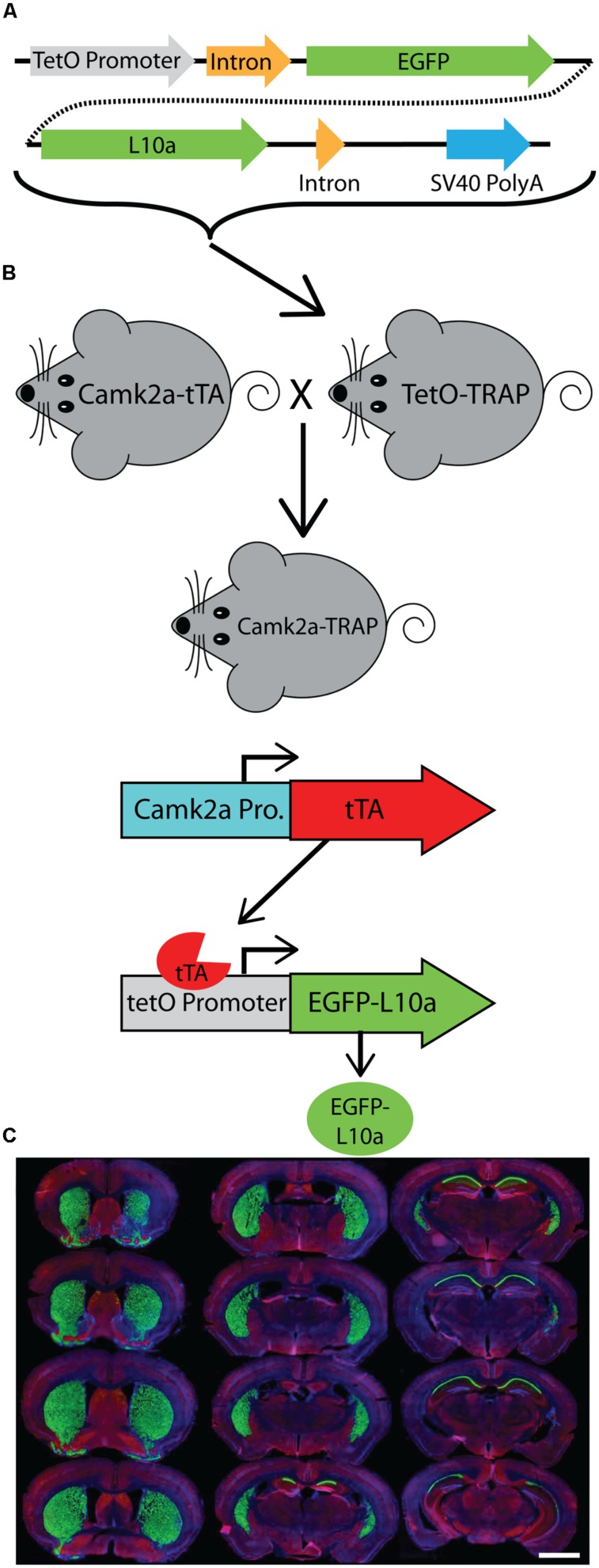
**Generation of a tetO driven EGFP-L10a transgenic mouse. (A)** Construct used to create the tetO-TRAP mouse line. **(B)** Model of the Camk2a-TRAP mouse. Camk2a-tTA mice were bred with tetO-TRAP mice to create Camk2a-TRAP mice. Expression of tTA under control of the Camk2a promoter leads to activation of the tetracycline response element (tetO) to drive expression of EGFP-L10a only in cells expressing Camk2a. **(C)** EGFP-L10a expression throughout the brain of a 3 month old Camk2a-TRAP mouse kept on food without doxycycline (dox) throughout life. Green, EGFP-L10a; red, Gad1; blue, dapi. Scale bar, 2 mm.

**FIGURE 2:**
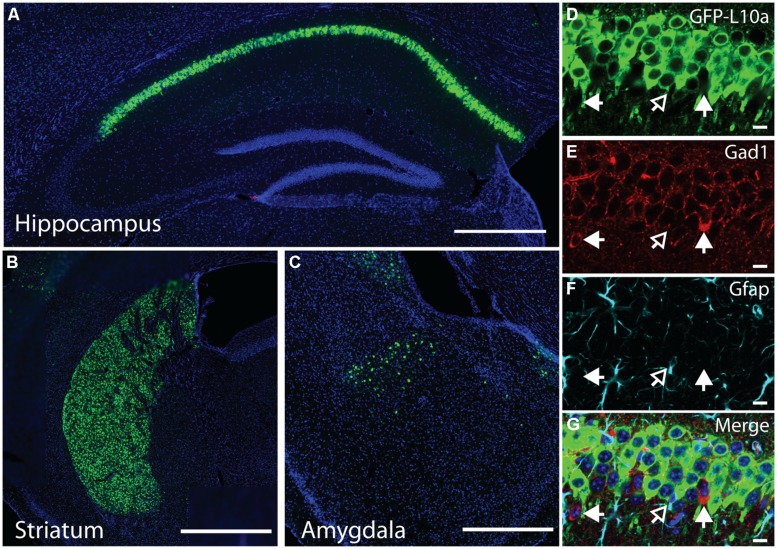
**EGFP-L10a expression in the Camk2a-TRAP mouse line. (A)** EGFP-L10a is highly expressed in pyramidal neurons of the CA1 region of the hippocampus. No EGFP-L10a is seen in the CA3 or dentate gyrus. Scale bar, 0.5 mm. **(B)** EGFP-L10a expression in medium spiny neurons of the striatum. Scale bar, 0.5 mm. **(C)** EGFP-L10a is sparsely expressed in projection neurons of the basal amygdala. Scale bar 0.5 mm. **(D–G)** EGFP-L10a is expressed exclusively in Camk2a expressing pyramidal neurons of the CA1 region of the hippocampus. No overlap of EGFP-L10a was observed with the interneuron marker Gad1, marked by filled in arrows **(E)**, or the astrocyte marker Gfap, marked by the opened arrow **(F)**. **(D)** EGFP-L10a, **(E)** Gad1, **(F)** Gfap, **(G)** Merge (including DAPI, blue). **(D–G)** scale bars, 10 μm.

Immediately following behavioral testing (Camk2a-TRAP mice), or on day 3 after FC (Fos-TRAP mice), mice were anesthetized with ketamine/xylazine and transcardially perfused with 0.1 M phosphate buffer followed by 4% paraformaldehyde (PFA) in 0.1 M phosphate buffer. Brains were removed and post-fixed in 4% PFA for 24 h. Brains were transferred to 30% sucrose for 48–72 hours, then snap frozen in isopentane. Coronal brain sections were sliced at 20 μm using a cryostat. Sections were stored at -20°C in cryoprotectant until use. Brain sections were washed in a solution of phosphate buffered saline (PBS) with 0.25% Triton-X-100 (PBS-T). Sections were then blocked at room temperature for 1 h in PBS-T containing 10% normal goat serum. Primary antibodies (Aves chicken anti-GFP 1:500, Millipore mouse anti-gad67 (Gad1) 1:10,000, Abcam rabbit anti-Gfap 1:2,000) were diluted in blocking solution and incubated overnight at 4°C. Specificity of all three primary antibodies was supported by the following three observations: (1) the staining patterns matched the known subcellular localization of the three proteins (extranuclear soma and processes), (2) morphology of the labeled cells, and (3) location of the labeled cells. After extensive washes with PBS-T, secondary antibodies (All from Jackson Immuno research; goat anti-chicken DyLight 488 1:500, goat anti-mouse cy3 1:2000, goat anti-rabbit DyLight 649 1:500) were diluted in blocking solution and applied to the sections for 2 h at room temperature followed by extensive PBS washes. Sections were stained with DAPI to visualize nuclei, mounted onto slides, coverslipped, and stored at 4°C. For **Figures 2D–G** and **5** a confocal laser-scanning microscope was used for image acquisition (Nikon-A1R). For **Figures 1C** and **2A–C** a slide scanner was used (Zeiss Axio Imager Z2 epifluorescence microscope with a Maerzhaeuser motorized scanning stage). For **Figure 4** a brightfield microscope was used (Nikon E800).

### IMMUNOPRECIPITATION AND mRNA ISOLATION

RNA was isolated using a modified version of the TRAP technique (Doyle et al., 2008; Heiman et al., 2008) that was optimized to remove background (Ainsley et al., 2014). Briefly, an anti-GFP antibody (HtzGFP-19C8) from the Monoclonal Antibody Core Facility at the Memorial Sloan-Kettering Cancer Center was covalently bound to magnetic epoxy beads (Invitrogen) followed by BSA treatment to reduce non-specific binding. Immediately following behavioral testing, Camk2a-TRAP mice were anesthetized using isoflurane. The hippocampi were rapidly dissected and rinsed in ice-cold dissection buffer (Heiman et al., 2008). Each hippocampus was added to 150 μl of homogenization buffer (Heiman et al., 2008) and homogenized using an automatic pestle. The tissue was centrifuged at 15,000 RPM for 10 min at 4°C. The lysate was transferred to a clean tube. For each sample, 1000 μg of protein was added to the prepared beads. Samples were incubated with the beads for 1 h at 4°C. The supernatant (SN) was saved for comparison to the immunoprecipitate (IP). After five washes with a KCl buffer (Heiman et al., 2008), RNA was extracted with Trizol LS. A back extraction was used to improve yield. Organic contaminants were removed with butanol and water-saturated diethyl ether washes (Krebs et al., 2009). RNA was precipitated using NaOAc, isopropanol, and linear acrylamide overnight at -80°C. After two washes with 80% EtOH, the RNA was resuspended in 15 μl nuclease-free water.

### CO-IMMUNOPRECIPITATION AND WESTERN BLOT

Protein immunoprecipitations were performed as described above for mRNA isolation. Before the start of the IP, 2.5% of the sample was removed and used as the input control. After the KCL buffer washes, the beads were saturated with sample buffer containing a reducing agent (Invitrogen). Samples were fractionated by gel electrophoresis using 4-12% Bis-Tris gels (Invitrogen), and transferred to nitrocellulose membranes (Invitrogen iBlot system). Membranes were blocked in 5% non-fat dried milk (NFDM) in TBST for 1 h and incubated in primary antibody diluted in 2% NFDM overnight at 4°C (HtzGFP-19C8 from the Monoclonal Antibody Core Facility at the Memorial Sloan-Kettering Cancer Center 1:500, Cell Signaling goat anti-mouse RPS6 1:1000, Millipore goat anti-mouse gapdh 1:20,000). The following day, membranes were washed in TBST and probed with secondary antibody for 1 h at room temperature (All from Jackson Immuno Research; donkey anti-mouse HRP, donkey anti-rabbit HRP). Immunoblots were visualized using Western Lighting®; Plus-ECL (PerkinElmer) according to manufacturer’s instructions.

### QUANTITATIVE RT-PCR AND BIOANALYZER ANALYSIS

All mRNA samples were DNase treated to remove any genomic DNA contamination by treatment for 30 min with TURBO^TM^ DNase (Ambion). The DNase was inactivated using a phenol–chloroform extraction. cDNA libraries were prepared using reverse transcription (RT) with Superscript III (Invitrogen) according to the manufacturer’s instructions, with the exception that both random hexamers and anchored-oligo dT primers were used during the RT reaction. Quantitative RT-PCR was performed with SYBR Green PCR master mix (Applied Biosystems) on the Mx4000 thermo cycler (Agilent) using the primer sequences listed in **Table 1**. The ΔΔct (ΔΔct = Δct1 – Δct2) was calculated from three biological replicates per primer set where Δct1 represented the normalized SN value to β-actin (Δct1 = gene SN ct – β-actin SN ct), and Δct2 represented the normalized IP value to β-actin (Δct2 = gene IP ct – β-actin IP ct). Bioanalyzer analysis was performed using the Agilent Technologies 2100 Bioanalyzer with RNA pico chips according to the manufacturers’ instructions.

**Table 1:** Primer sequences used for quantitative RT-PCR.

Gene name	Primer sequence
Gad1_F	5′-CACAGGTCACCCTCGATTTTT-3′
Gad1_R	5′-ACCATCCAACGATCTCTCTCATC-3′
Pkia_F	5′-TCGCAGCCACGGGTGAAACG-3′
Pkia_R	5′-TCCAAGCACAGCCCAGGTGA-3′
Gfap_F	5′-CGGAGACGCATCACCTCTG-3′
Gfap_R	5′-AGGGAGTGGAGGAGTCATTCG-3′
Camk2a_F	5′-TTTGAGGAACTGGGAAAGGG-3′
Camk2a_R	5′-CATGGAGTCGGACGATATTGG-3′
Naa38_F	5′-GGCTGTTATTACTTCTGATGGCA-3′
Naa38_R	5′-ACACCACTTGTTCTACTCCCT-3′
β-actin_F	5′-GGCTGTATTCCC CTC CATCG-3′
β-actin_R	5′-CCAGTTGGTAACAATGCCATGT-3′

## RESULTS

### GENERATION OF A MOUSE LINE THAT EXPRESSES EGFP-L10a UNDER CONTROL OF THE tetO PROMOTER

We generated a transgenic mouse expressing the ribosomal protein L10a tagged with EGFP under the control of the tetracycline operon (tetO-TRAP; **Figure 1A**, see Materials and Methods). After screening multiple founder lines, one line was selected for further use. To test the ability of our tetO-TRAP mouse line to express EGFP-L10a in a tTA-dependent manner, we crossed the tetO-TRAP mouse with a Camk2a-tTA mouse to create a mouse that expresses EGFP-L10a under the Camk2a promoter (Camk2a-TRAP; **Figure 1B**). Previous work using the same Camk2a-tTA drive line resulted in strong transgene expression in the CA1 region of the hippocampus (but not CA3 or dentate gyrus) as well as strong expression throughout the striatum (Mayford et al., 1996). Immunohistochemical analysis of brain sections from 3 month old Camk2a-TRAP mice revealed a similar expression pattern (**Figure 1C**). The CA1 region of the hippocampus showed strong expression of EGFP-L10a, while the CA3 and dentate gyrus did not express EGFP-L10a (**Figure 2A**). Strong EGFP-L10a expression was also observed in the striatum, and appeared restricted to medium spiny neurons that are known to express Camk2a (**Figure 2B**). In addition, analysis of the amygdala revealed sparse EGFP-L10a expression in the basal subdivision, presumably in projection neurons known to express Camk2a (**Figure 2C**). The lack of EGFP-L10a expression in other types of neurons known to express Camk2a is due to a more restricted expression of tTA, presumably due to the genomic integration site of the Camk2a-tTA transgene (Mayford et al., 1996). Expression of EGFP-L10a in Camk2a-TRAP mice was tTA-dependent, as single transgenic tetO-TRAP mice did not express any EGFP-L10a in the brain (data not shown). In order to confirm the cell-type specificity of EGFP-L10a expression in the Camk2a-TRAP mice, we performed immunostaining for Gad1, a marker of inhibitory interneurons, as well as Gfap, an astrocyte marker. No overlap of EGFP-L10a with either Gad1 or Gfap was seen in the CA1, confirming that the transgene was not expressed in CA1 interneurons or astrocytes (**Figures 2D–G**). Compared with our recent study (Ainsley et al., 2014), the expression data in **Figures 1** and **2** extend the analysis of EGFP-L10a expression patterns in Camk2a-TRAP mice by providing more detail for a larger number of brain regions. In summary, the expression data from Camk2a-TRAP mice confirm cell-type specific tTA-dependent expression of EGFP-L10a, thereby validating proper transgene functioning in the tetO-TRAP mouse line.

### COLLECTION OF RIBOSOME-BOUND mRNA FROM CA1 PYRAMIDAL NEURONS IN THE Camk2a-TRAP MOUSE

**FIGURE 3:**
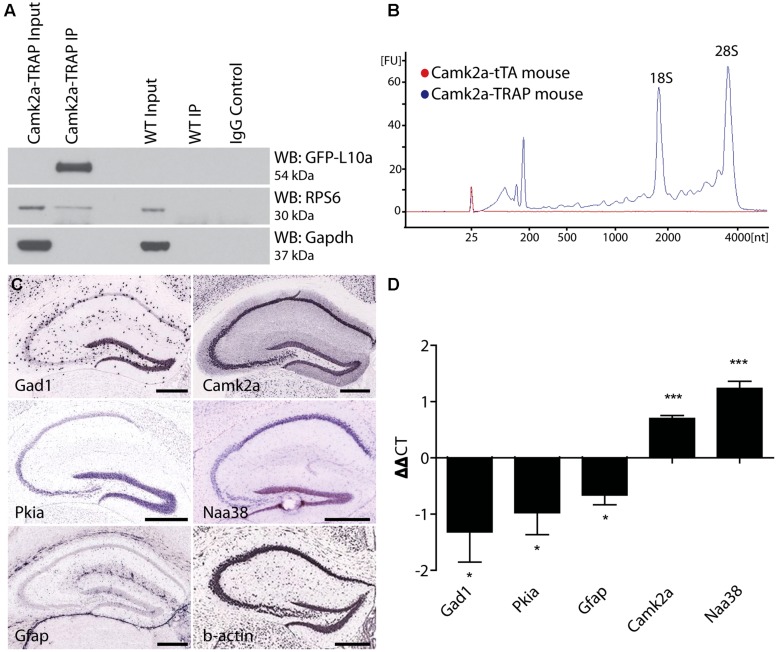
**Cell-type specific collection of ribosome-bound mRNA using the Camk2a-TRAP mouse. (A)** Representative affinity purification of EGFP-L10a and co-immunoprecipitation of the small ribosomal protein RPS6 from whole brain lysate of Camk2a-TRAP animals (*N* = 6), and wild type littermate controls (WT; *N* = 6). 2.5% of sample was used for input prior to the IP. EGFP-L10a is only visible in the IP lane of the Camk2a-TRAP mice because the IP samples were highly concentrated compared to the input lanes. No RPS6 was co-purified from wild type samples that did not contain EGFP-L10a. The IgG control lane confirms the absence of IgG contamination artifacts. **(B)** Representative bioanalyzer plot of RNA samples from Camk2a-TRAP (blue; RIN mean ± SEM, 7.48 ± 0.14, *N* = 7) and single transgenic littermates (red; RIN mean ± SEM, 2.2 ± 1.2, *N* = 3) indicating successful isolation of ribosome-bound RNA from Camk2a-TRAP mice, but not single transgenic controls. mRNA is represented by the area under the curve between 200 and 4000 nt, with the 18S and 28S peaks representing rRNA present in the affinity purified ribosomes. The peaks between 25 and 200 nt represent small rRNAs and tRNAs, as well as highly degraded mRNA typically seen with Trizol extraction protocols. **(C)** *In situ* hybridization images from the Allan Brain Atlas showing localization of genes used for qPCR analysis. Two negative control genes (Gad1 and Gfap) are not expressed in CA1 pyramidal neurons, and one negative control gene (Pkia) is mainly expressed outside of CA1 pyramidal neurons. Positive control genes (Camk2a and Naa38) are widely expressed in CA1 pyramidal neurons. Beta-actin is expressed in all cell-types throughout the hippocampus and was therefore used as a housekeeping gene for ΔΔct analysis. Scale bars, 0.42 mm. **(D)** qPCR analysis of mRNA collected with the TRAP protocol from Camk2a-TRAP animals. Negative control genes (Gad1, Pkia, and Gfap) all showed a significant depletion from the IP samples, while positive control genes (Camk2a and Naa38) both showed significant enrichment in the IP samples. (One tailed *t*-test with mu = 0. *P < 0.05, ***P < 0.001; Gad1 *P* = 0.046, Pkia *P* = 0.045, Gfap *P* = 0.016, Camk2a *P* = 0.0005, Naa38 *P* = 0.001). *N* = 3.

To ensure that the EGFP-L10a expressed by Camk2a-TRAP mice integrates into ribosomal complexes, we performed a Co-IP of EGFP-L10a, and probed for a member of the small ribosomal subunit, RPS6. Co-IP of RPS6 would indicate that EGFP-L10a had successfully integrated into the large ribosomal complex, and that this large ribosomal complex was subsequently able to associate with the small ribosomal complex to form a complete ribosome. Immunoprecipitation of EGFP-L10a from whole brain lysate resulted in the Co-IP of RPS6, indicating functional integration of EGFP-L10a into whole ribosomes (**Figure 3A**). RPS6 was not Co-IPed when using a single transgenic animal that did not express EGFP-L10a, confirming the specificity of the Co-IP.

The immunohistochemical analysis of Camk2a-TRAP mice revealed that EGFP-L10a is expressed in excitatory pyramidal cells of the CA1, but not in interneurons or astrocytes in the CA1 or in other subregions of the hippocampus (**Figure 2**). To determine if this expression pattern enabled the collection of cell-type specific mRNA from CA1 pyramidal neurons, we attempted to collect ribosome-bound mRNA from whole hippocampal lysate of Camk2a-TRAP mice. In a previous study we found that neuronal activation increased the association of mRNA with ribosomes (Ainsley et al., 2014), in agreement with previous reports (Weiler and Greenough, 1991, 1993; Job and Eberwine, 2001; Niere et al., 2012; Buxbaum et al., 2014). We therefore subjected all of our animals to a 500 s long contextual FC trial before tissue collection. The quality of the ribosome-bound mRNA isolated from Camk2a-TRAP mice was analyzed using an RNA Bioanalyzer. FC single transgenic Camk2a-tTA animals subjected to the TRAP IP were used as negative controls. Bioanalyzer analysis of Camk2a-TRAP RNA samples revealed the presence of high-quality RNA as indicated by a high RNA integrity number (RIN). The RIN value is calculated based on the entire electrophoretic trace of the RNA sample, and takes into account the quantity of RNA degradation (Mueller et al., 2004). No RNA was detected in TRAP IP samples from single transgenic mice, confirming the specificity of our TRAP IP protocol (**Figure 3B**). The Camk2a-TRAP RNA sample contained both rRNA and mRNA. This confirmed that the TRAP IP collected ribosomes (which contain rRNA) bound to mRNA, in agreement with the incorporation of EGFP-L10a into functional translating ribosomes.

Within the hippocampus, EGFP-L10a is only expressed in CA1 pyramidal neurons, so we expected to see an enrichment of transcripts expressed in CA1 pyramidal neurons in the TRAP IP samples, as well as a depletion of genes expressed completely or predominantly outside of CA1 pyramidal neurons. We therefore performed a qPCR analysis of two genes not expressed in CA1 pyramidal neurons (Gad1 and Gfap), one gene expressed predominantly outside of CA1 pyramidal neurons (Pkia), and two genes that are widely expressed in CA1 pyramidal neurons (Camk2a and Naa38; **Figure 3C**; Lein et al., 2007). ΔΔct values were calculated using β-actin as a reference gene. Only the two genes known to be widely expressed in CA1 pyramidal neurons were enriched in the IP samples, while the other genes showed a marked depletion (**Figure 3D**). In summary, our data show that Camk2a-TRAP mice enable the cell-type specific collection of ribosome-bound mRNA from CA1 pyramidal neurons.

### CREATION OF AN ACTIVITY-INDUCED EGFP-L10a MOUSE LINE

After validation of the Camk2a-TRAP mouse line, we used the tetO-TRAP mouse to create a second TRAP mouse model that enables the activity-induced expression of EGFP-L10a. For this purpose, we crossed the tetO-TRAP mouse line with a Fos-tTA mouse to drive expression of EGFP-L10a under control of the activity-regulated Fos promoter (Fos-TRAP mouse). Fos is an immediate early gene (IEG) whose promoter is rapidly activated upon neuronal stimulation (Flavell and Greenberg, 2008). Accordingly, the Fos-tTA mouse can trigger expression of tetO-regulated reporter proteins in activated neurons, including activated CA1 pyramidal neurons (Reijmers et al., 2007; Matsuo et al., 2008; Tayler et al., 2013). The Fos-tTA mouse is also referred to as the TetTag mouse, which stands for “Tet”racycline controlled “Tag”ging. The time-window during which tagging of activated neurons occurs is opened by the removal of dox from the food, after which Fos-promoter driven tTA can bind to the tetO promoter. The time-window is closed by adding dox back to the food, after which dox prevents binding of tTA to the tetO promoter (**Figure 5A**).

**FIGURE 4:**
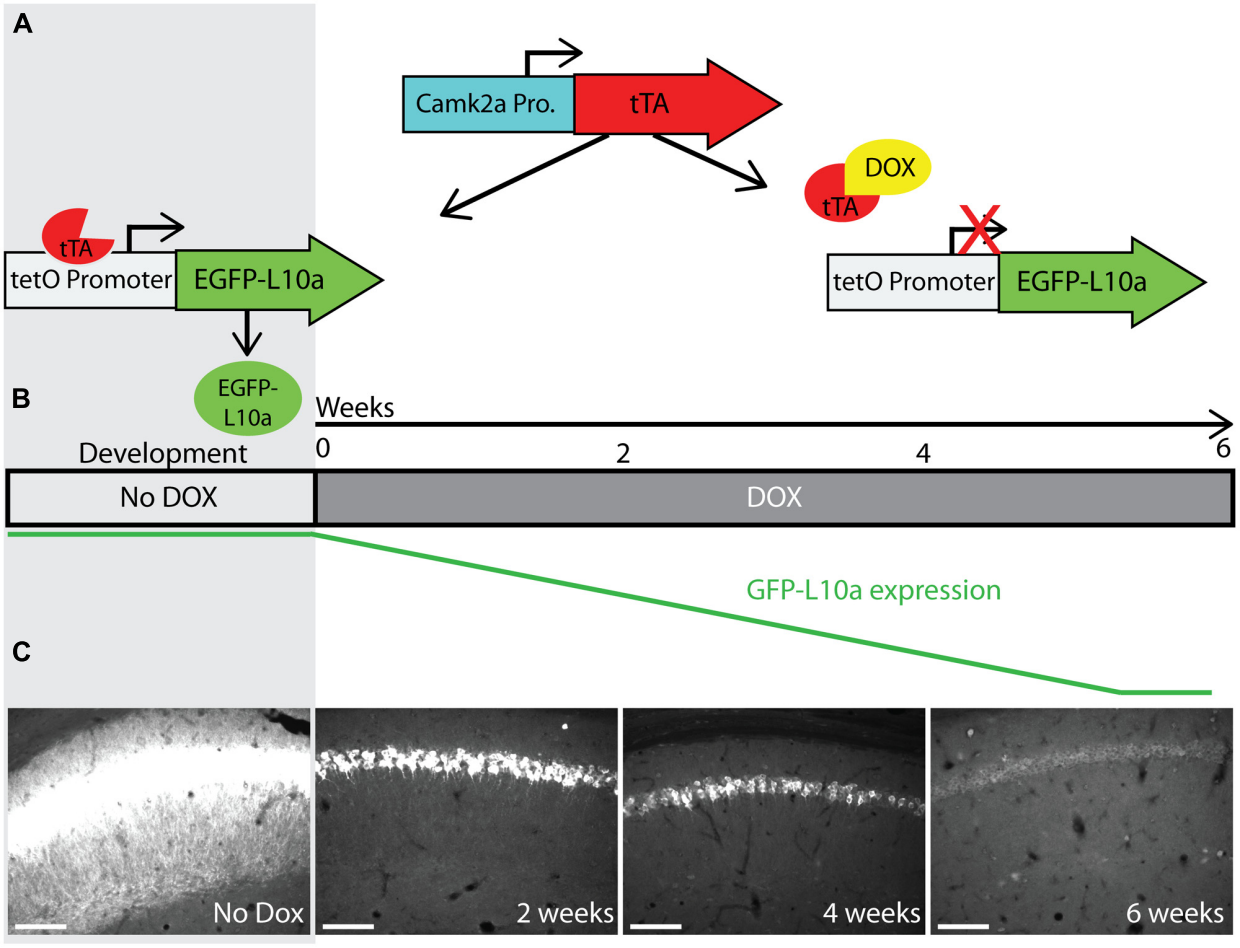
**Complete degradation of developmentally expressed EGFP-L10a requires 6 weeks. (A)** Schematic representation of dox control over EGFP-L10a expression in the Camk2a-TRAP mouse model. In the absence of dox, tTA is able to bind to the tetO promoter and drive EGFP-L10a expression. In the presence of dox, tTA is no longer able to activate the tetO promoter, thereby preventing any further expression of EGFP-L10a. **(B)** Experimental design showing the time-points of brain dissection after adding dox, and the anticipated level of EGFP-L10a protein. **(C)** Images of the CA1 region of the hippocampus show that depletion of high levels of EGFP-L10a takes 6 weeks of continuous dox. Scale bar, 100 μm. *N* = 1 for each time-point.

Successful mRNA isolation by TRAP requires high expression levels of EGFP-L10a, since this fusion protein has to out-compete endogenous L10a for its incorporation into ribosomes. However, the Fos promoter is activated for only a short period of time after neuronal activation. We were concerned that the brief period of Fos promoter activation would not drive sufficient EGFP-L10a expression for successful mRNA isolation using the TRAP technique. A previous study characterizing tetO-driven transgene expression found that mice bred and raised in the absence of dox later showed higher expression levels of the transgene when compared to mice bred and raised in the presence of dox (Bejar et al., 2002). One explanation for this phenomenon is that the expression of the tetO-driven transgene during brain development might prevent its epigenetic silencing. To prevent potential epigenetic silencing, we bred and raised Fos-TRAP mice in the absence of dox, put the mice on food with dox after weaning, and then waited until all the developmentally expressed EGFP-L10a was degraded. For this, we first needed to determine for how long Fos-TRAP mice had to be kept on food with dox in order to achieve complete degradation of EGFP-L10a. We found that a period of 6 weeks of dox following high developmental EGFP-L10a expression can be sufficient for the complete degradation of EGFP-L10a (**Figures 4A–C**).

**FIGURE 5:**
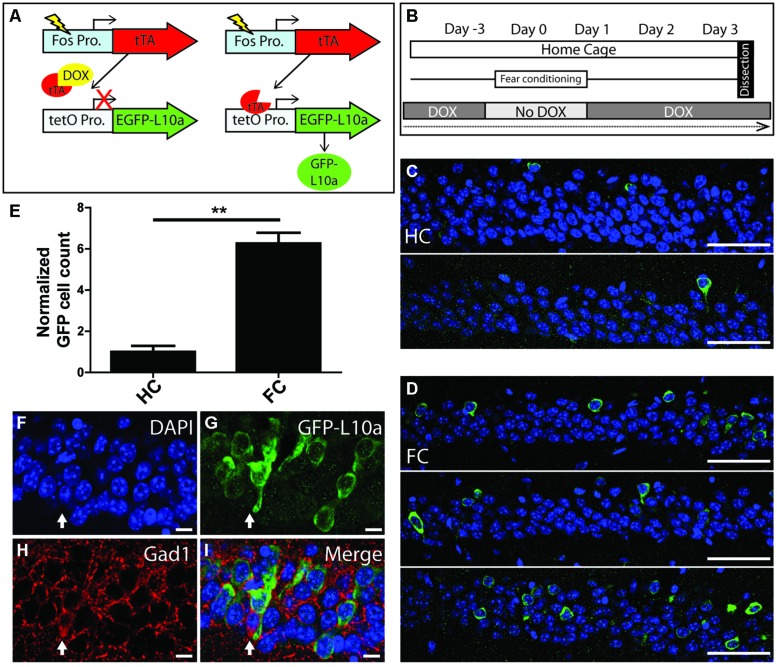
**Creation and characterization of the Fos-TRAP mouse line. (A)** Schematic of the Fos-TRAP mouse model. Activation of the activity induced Fos promoter induces tTA transcription. When present, dox binds to tTA and prevents it from interacting with the tetO promoter. Removal of dox enables tTA to bind to the tetO promoter and drive expression of EGFP-L10a. **(B)** Experimental design. Mice were given dox chow for 8–10 weeks after weaning to enable complete degradation of developmentally expressed EGFP-L10a. On day-3, dox was removed from the diet. FC was done in the absence of dox on day 0 to allow EGFP-L10a expression in activated neurons. Following FC, all animals were put back on dox to prevent further EGFP-L10a transcription. Brains were collected on day 3 to allow time for EGFP-L10a protein accumulation. **(C,D)** EGFP-L10a expression in pyramidal cells of the CA1 region of the hippocampus in **(C)** home cage (HC, *N* = 2) Fos-TRAP mice, and **(D)** fear conditioned (FC, *N* = 3) Fos-TRAP mice. Scale bars, 50 μm. **(E)** Quantification of EGFP-L10a positive cells per area of CA1 in HC (*N* = 2) and FC (*N* = 3) mice. All values were normalized to a HC group average of 1. **P < 0.01. Two tailed *t*-test *P* = 0.0049. **(F–I)** EGFP-L10a expression in the CA1 of Fos-TRAP mice was restricted to pyramidal cells. Mice were bred and raised off dox to allow for maximal EGFP-L10a expression. No overlap of EGFP-L10a was observed with the interneuron marker Gad1, marked by the filled in arrow **(H)**. Scale bars, 10 μm. **(F)** Dapi, **(G)** EGFP-L10a, **(H)** Gad1, **(I)** merge.

To determine if the Fos-TRAP mouse line can be used for the activity-induced expression of EGFP-L10a, we compared EGFP-L10a expression in HC animals versus animals that underwent a FC trial. Fos-TRAP mice were bred and raised without dox to prevent potential epigenetic silencing, and were kept on dox for at least 6 weeks following weaning to allow complete degradation of all developmentally expressed EGFP-L10a. We removed dox from the diet of both FC and HC animals on day-3 of the experiment, and subjected the FC group to FC on day 0 (**Figures 5A,B**). At the end of day 0, both HC and FC mice were put back on dox, and brains were dissected on day 3. Comparison of the HC and FC animals revealed a significant increase in the number of EGFP-L10a expressing cells in the CA1 of the hippocampus of the FC group as compared with the HC group (**Figures 5C–E**), in agreement with an earlier study that used Fos-tTA × tetO-histoneGFP mice (Tayler et al., 2013). Therefore, the increased neuronal activation in the CA1 caused by FC resulted in the predicted increase in EGFP-L10a expression. In addition, analysis of cell-type specificity of EGFP-L10a expression found no overlap of EGFP-L10a with the interneuron marker Gad1, revealing that EGFP-L10a expression in the CA1 is specific to pyramidal neurons (**Figures 5F–I**). The Fos-TRAP mouse can therefore be used to drive EGFP-L10a expression in CA1 pyramidal neurons that are activated during a behavioral test.

## DISCUSSION

In order to expand the arsenal of TRAP-based tools for isolating ribosome-bound mRNA from defined cell types, we have generated a novel mouse line in which EGFP-L10a expression is under the control of the tetO promoter. We crossed this line with two different transgenic tTA mice, one driving tTA under the control of the Camk2a promoter, and one driving tTA under control of the Fos promoter to create Camk2a-TRAP and Fos-TRAP mice, respectively. We found that in both lines EGFP-L10a exhibited expression patterns similar to other tetO-driven genes that have previously been crossed into Camk2a-tTA and Fos-tTA driver mice. Using the Camk2a-TRAP mice we showed that tetO-driven EGFP-L10a integrates into functional ribosomes, and that high quality cell-type specific mRNA can be collected through affinity purification of the EGFP-L10a tagged ribosomes.

By using the tTA system we enabled both spatial and temporal control over EGFP-L10a expression. The ability to cross the tetO-TRAP mouse line with various cell-type specific tTA driver lines, such as the Camk2a-tTA line, confers spatial control by restricting EGFP-L10a expression to these cell-types. Though a number of BAC transgenic TRAP mice have been generated for the targeting of various cell-types in the brain (Doyle et al., 2008), our tetO-TRAP mouse significantly expands the number of cell types that are now amendable to TRAP analysis. The ability to manipulate the timing of EGFP-L10a expression through the use of dox in the food confers temporal control. Temporal control allows the use of activity-induced promoters such as the Fos promoter to tag cells that are activated during a specific time window, for example when a behavioral task is executed. In addition, temporal control enables more specific expression patterns when using promoters that drive differential expression patterns during different phases of development.

In this paper, we report how the Camk2a-TRAP mouse can be used to collect ribosome-bound mRNA from complete CA1 pyramidal neurons by using a straightforward protocol during which the whole hippocampus is dissected, homogenized, and subjected to TRAP IP. By using this protocol, we were able to collect high quality, ribosome-bound mRNA from CA1 pyramidal neurons at a defined time-point immediately following behavioral activation. In addition to this whole hippocampal dissection protocol, we reported in a previous paper that the Camk2a-TRAP mouse can be used to collect ribosome-bound mRNA from *in vivo* dendrites of CA1 pyramidal neurons by using a more specific dissection protocol (Ainsley et al., 2014). To the best of our knowledge, the Camk2a-TRAP mouse is the first reported mouse line that enables the collection of ribosome-bound mRNA from CA1 pyramidal neurons. Given the important role of CA1 pyramidal neurons in learning and memory, we anticipate that future studies will use the Camk2a-TRAP mouse to discover and characterize molecular mechanisms of cognition.

The Fos-TRAP mouse is the first mouse model designed for the collection of ribosome-bound mRNA specifically from neurons that are activated during a behavioral test. Thereby, the Fos-TRAP mouse should make it possible to study protein synthesis in neurons that are functionally defined. Though our initial data support the feasibility of using the Fos-TRAP mouse for these purposes, two caveats need to be addressed in future studies. First, the mRNA isolation experiment reported in this paper was done using the Camk2a-TRAP mouse line, which expresses high EGFP-L10a levels throughout its lifetime. Because of the inducible nature of the Fos-TRAP mouse line, EGFP-L10a is only expressed during a short time window. It remains to be determined whether the resulting EGFP-L10a expression levels will produce a sufficient number of EGFP-tagged ribosomes to enable successful affinity purification. Second, after translation, EGFP-L10a must integrate into functional ribosomes in order for the TRAP IP protocol to be effective. Therefore, sufficient time must be allowed after Fos promoter activation for EGFP-L10a translation and ribosome integration. At this point, it is unknown how long it takes for a newly synthesized EGFP-L10a protein to be incorporated into a functional ribosome. Though these caveats need to be addressed in future studies, our current data provide an important first validation by showing that EGFP-L10a levels in CA1 pyramidal neurons of Fos-TRAP mice increase after FC. In addition, the creation of the Fos-TRAP mouse line affirms the versatility of the tetO-TRAP mouse by showing that it can be crossed with different tTA drivers to enable a variety of EGFP-L10a expression patterns.

In summary, we developed a tetO-TRAP mouse line for expressing EGFP-L10a with high spatial and temporal control, thereby expanding the collection of available TRAP mouse lines. Compared with other methods for collecting cell-type specific mRNA, the TRAP technique is more efficient than LCM and manual cell sorting, causes less gene expression artifacts than FACS and other cell sorting methods that require dissociation of cells from tissue, and collects translated mRNA instead of total mRNA. We have demonstrated that the tetO-TRAP mouse can be crossed with both the Camk2a-tTA and Fos-tTA driver lines to produce cell-type specific EGFP-L10a expression, and that it can be used for collecting ribosome-bound mRNA from CA1 pyramidal neurons. Although our analyses were focused on CA1 pyramidal neurons, we anticipate that the tetO-TRAP mouse can be used for a large variety of translational profiling purposes, both within the brain and in peripheral tissues, by crossing the tetO-TRAP mouse with other tTA driver mice (Schonig et al., 2010).

## Conflict of Interest Statement

The authors declare that the research was conducted in the absence of any commercial or financial relationships that could be construed as a potential conflict of interest.
